# A flexible MOF formed from Cu^II^ and 2,3-di­hydroxy­terephthalic acid

**DOI:** 10.1107/S205698902500653X

**Published:** 2025-07-29

**Authors:** Russell M. Main, Aidan P. McKay, Russell E. Morris

**Affiliations:** a EaStCHEM School of Chemistry, Purdie Building, North Haugh, St Andrews KY16 9ST, United Kingdom; University of Buenos Aires, Argentina

**Keywords:** metal–organic framework, flexible MOF, SCXRD, crystal structure

## Abstract

Metal–organic frameworks (MOFs) are an exciting class of porous materials with applications in many fields. To find the optimal MOF for every application it is important to synthesize new materials to find those with the desirable properties. Here we present a MOF based on Cu^II^ and 2,3-di­hydroxy­terephthalate, whose structure in both fully solvated and partially desolvated forms, namely, poly[[tri­aqua­(μ-2,3-di­hydroxy­terephthalato)(μ-2,3-dioxido­terephthalato)tricopper(II)] monohydrate], {[Cu_3_(C_8_H_2_O_6_)(C_8_H_4_O_6_)(H_2_O)_3_]·H_2_O}_*n*_, and poly[[di­aqua­(μ-2,3-di­hydroxy­terephthalato)(μ-2,3-dioxidoterephthalato)tricopper(II)] ethanol monosolvate monohydrate] {[Cu_3_(C_8_H_2_O_6_)(C_8_H_4_O_6_)(H_2_O)_2_]·C_2_H_5_OH·H_2_O}_*n*_, has been solved with single-crystal X-ray diffraction. It shows one-dimensional hexa­gonal channels and a flexible behaviour in response to changes in solvation.

## Abstract 2

The crystal structures of a flexible metal–organic framework based on Cu^II^ and 2,3-dhtp in different states of solvation are reported.

## 1. Chemical context

Metal-organic frameworks (MOFs) are a rapidly growing class of porous materials (Ettlinger *et al.*, 2024 ▸). They consist of metal ions or clusters bound together by organic linkers to form crystalline frameworks with potential porosity (Batten *et al.*, 2013 ▸). The discovery of new MOFs is important, not only for the academic pleasure of synthesising new materials, but also to allow for advancements across many fields, from the adsorption of gases to zygote gene therapy (Martínez-Ahumada *et al.*, 2020 ▸; Li *et al.*, 2011 ▸; Gonzalez *et al.*, 2017 ▸; Sameni *et al.*, 2024 ▸; Howarth *et al.*, 2017 ▸; Xu & Yaghi, 2020 ▸). There are various methods for generating new MOFs. These include using reticular chemistry, to modify known topologies to modify pore size or reactivity (Freund *et al.*, 2021 ▸), and mixing new combinations of ions and linkers to generate completely new systems with different structures and properties (Stock & Biswas, 2012 ▸). Both techniques are important, and it is often a combination of the two that leads to the optimal material for any one application.

The latest generation of MOFs includes those with flexible or responsive characteristics (Kitagawa, 2017 ▸). Capable of responding to external stimuli such as heat, solvent or pressure these materials show great potential in many fields, for instance in separations (Schneemann *et al.*, 2014 ▸). When searching for flexible MOFs it is important to consider both the metal, and its chemical lability, as well as the geometry of the linker. The Cu^II^ ion is a particularly labile one and well suited to generating flexible MOFs (Rieth *et al.*, 2019 ▸; McHugh *et al.*, 2018 ▸). The linker can also be responsible for flexibility by being flexible itself or by its structure frustrating the binding around the metal site, allowing for flexible behaviour in the system as a whole (Schneemann *et al.*, 2014 ▸).

Understanding the structures of MOFs is important for knowing how best to apply a particular framework. This is especially true when the structure changes in response to external stimuli. Single crystal X-ray diffraction (scXRD) is one the most powerful tools in the modern chemist’s arsenal for understanding the structure of a material. Here we present a new MOF made from Cu^II^ and 2,3-di­hydroxy­terephthalic acid (2,3-dhtp). By using scXRD we have determined the structure as well as the structure after partial desolvation, with the MOF showing a flexible response to this change.
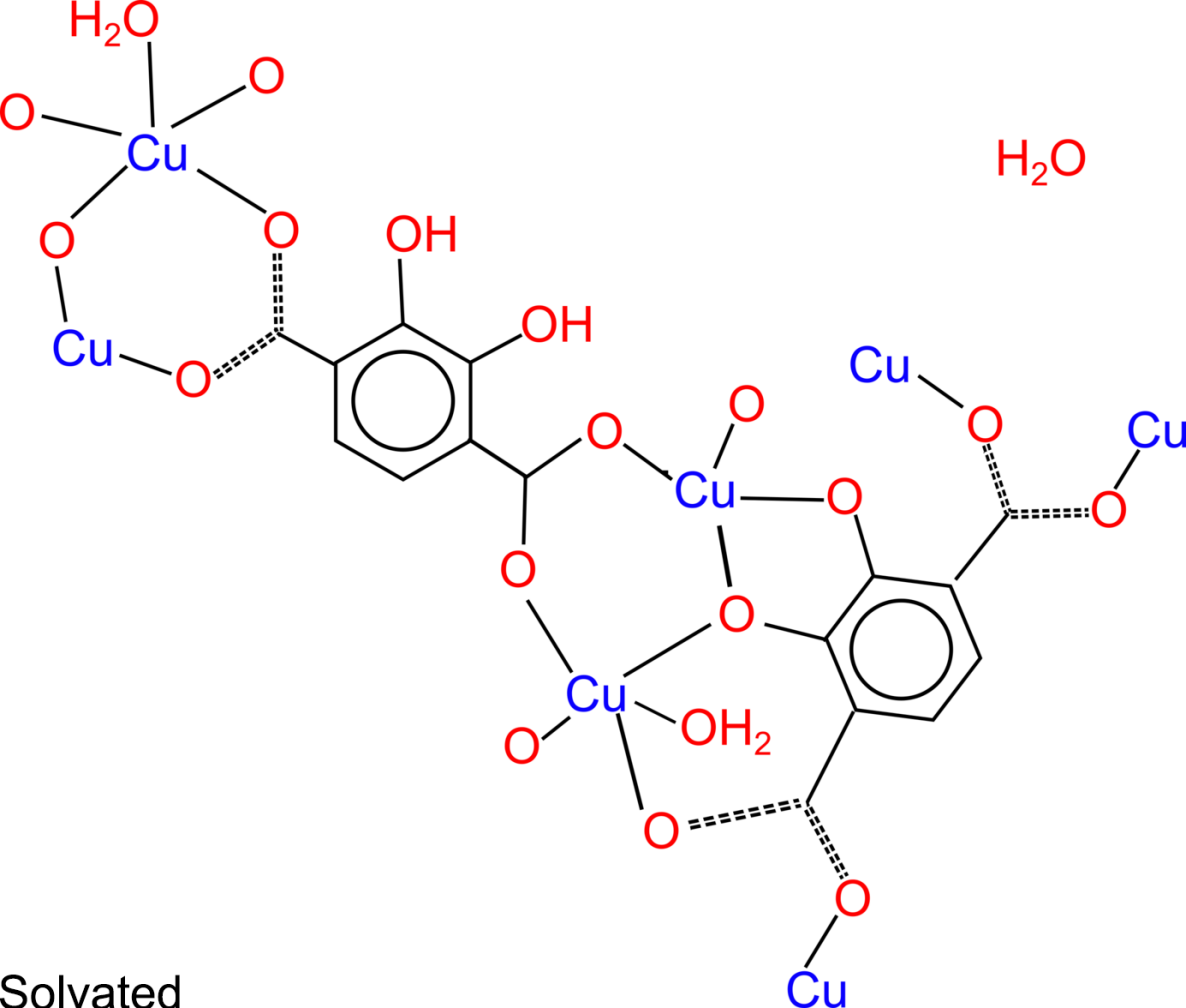

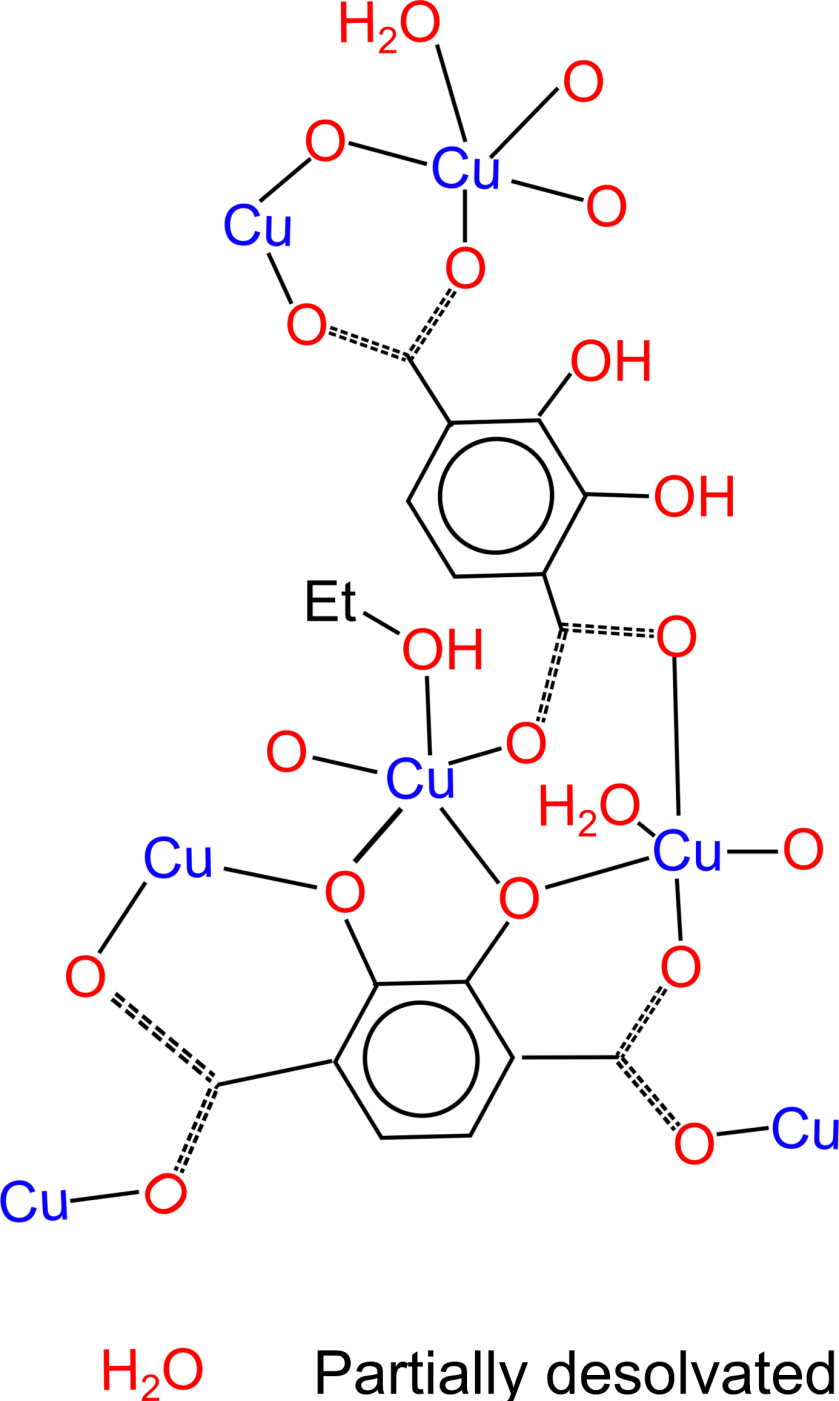


## 2. Structural commentary

Reacting copper acetate and 2,3-dhtp in a 1:1 DMF:water solvent mix at room temperature produces brown crystals of a product denoted St Andrews MOF (SIMOF-6), which were suitable for single crystal X-ray crystallography. The structure crystallized in the ortho­rhom­bic *Pnma* space group with unit-cell parameters *a* = 26.9440 (5) Å, *b* = 16.4946 (3) Å, *c* = 6.8910 (2) Å, and cell volume 3062.5 (1) Å^3^. It has formula [Cu_2_(C_8_H_2_O_6_)(C_8_H_4_O_8_)(H_2_O)_2_]·(H_2_O)·(solvent) with an estimated additional five water solvates. There are two distinct Cu sites, the first (Cu1) is five-coordinate distorted square pyramidal with four bonds in a plane to 2,3-dhtp mol­ecules [1.922 (3)–1.961 (2) Å] and one perpendicular to a water ligand [2.257 (3) Å]. The second site is also five-coordinate distorted square pyramidal, with four bonds in a plane to 2,3-dhtp mol­ecules [1.952 (4)–1.989 (5) Å]. It is, however, disordered above and below this plane with the copper modelled in across two sites with 56:44 occupancy. Each site is bonded to a water ligand [2.31 (2) and 2.29 (2) Å] perpendicular to the 2,3-dhtp plane and with a water solvate in the opposing position which is hydrogen bonded to the copper bound water ligands (Fig. S1*a*). One 2,3-dhtp ligand is tetra-anionic with both hydroxyl groups coordinating as well as the carboxyl­ates, while the second independent ligand is di-anionic and coordinates through the carboxyl­ates with the position of the hydroxyl groups disordered 50:50.

**Figure 1:**
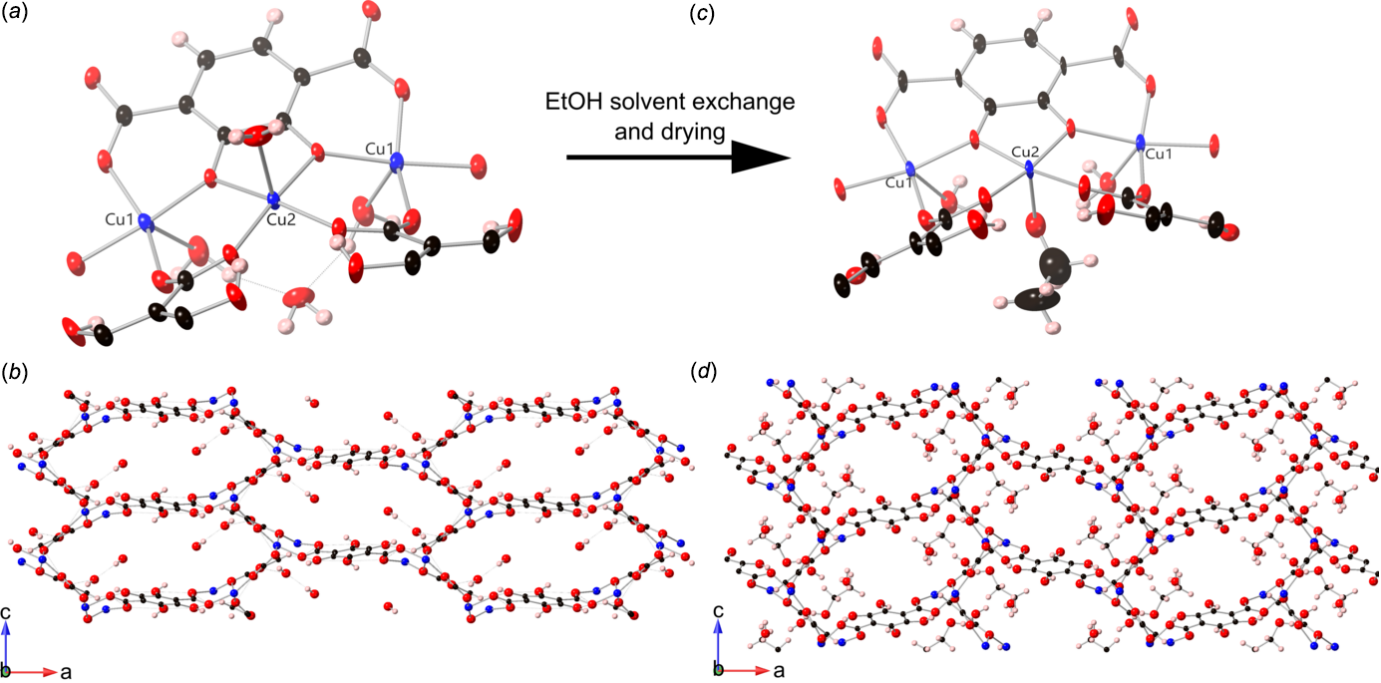
(*a*) 50% probability ellipsoids showing the SBU of solvated SIMOF-6. (*b*) Ball and stick model of solvated SIMOF-6 viewed down the crystallographic *b* axis. (*c*) 50% probability ellipsoids showing the SBU of partially desolvated SIMOF-6. (*d*) Ball and stick model of partially desolvated SIMOF-6 viewed down the crystallographic *b* axis. H = pink, O = red, C = black, Cu = blue, some disorder modelling has been removed for clarity.

The SIMOF-6 structure contains a planar secondary building unit (SBU) in which three Cu atoms sit in a plane bonded to a 2,3-dhtp mol­ecule with a 4^−^ charge (Fig. 1 ▸*a*). The middle Cu atom is disordered and five-coordinate (Cu2) while the outer two are ordered five-coordinate (Cu1). There are two bridging disordered 2,3-dhtp mol­ecules coming from each SBU in which the phenol groups are protonated giving them a 2^−^ charge. The disorder in these linkers consists of the 2,3-dhtp being in two orientations a 180° rotation apart so that the phenol groups appear on both sides of the ring with 50% occupancy. The remaining bonding of the SBU is to the 2,3-dhtp carboxyl­ates of SBUs on either side. This binding produces 1D chains of Cu atoms and 4^−^ 2,3-dhtp running down the crystallographic *b*-axis direction, linked together by the disordered 2^−^ 2,3-dhtp to produce 1D hexa­gonal channels running down the *b*-axis direction (Fig. 1 ▸*b*).

This material can be partially desolvated through solvent exchange with ethanol (by washing on filter) and drying at atmospheric pressure and a temperature of 333 K overnight. This process caused some damage to the crystals but they remained suitable for X-ray analysis. The partially desolvated structure stayed in the *Pnma* space group but the unit cell shrank to *a* = 23.412 (2) Å, *b* = 16.7735 (8) Å, *c* = 7.4097 (5) Å and the unit-cell volume became 2909.9 (3) Å^3^. The formula becomes [Cu_2_(C_8_H_2_O_6_)(C_8_H_4_O_8_)(C_2_H_5_O)(H_2_O)]·H_2_O·(solvent) with the overall structure similar to the solvated form with two distinct Cu sites which are both five-coordinate distorted square pyramidal. The first site, Cu1, contains four bonds in a plane to 2,3-dhtp mol­ecules [1.920 (7)–1.961 (6) Å] and one perpendicular to a water ligand [2.256 (7) Å] that lies in the ‘wall’ of the pore. The second site, Cu2, has four bonds in a plane to 2,3-dhtp mol­ecules [1.945 (6)–1.950 (6) Å] and one perpendicular to a solvent mol­ecule [2.304 (12) Å] that lies in the pore. There is no sign from this data of the copper disorder seen in the solvated form (Fig. S1*b*). The binding of the SBUs is still relatively unchanged (Fig. 1 ▸*c*). However, the small changes around the SBU, caused by loss of solvent, have caused the distorted hexa­gonal channels to be slightly offset so that the Cu chains are closer together and the disordered 2^−^ 2,3-dhtp is rotated, leading to a slightly smaller pore and more densely packed structure (Fig. 1 ▸*d*).

No further solvent in either structure could be reasonably modelled, so both were treated with a mask with a 1.2 Å probe. In the solvated structure, the mask found 316 electrons per unit cell within the 1226 Å^3^ of free pore volume, which equates to approximately 30 additional water mol­ecules per unit cell. However, from the reaction conditions it is possible that some of this density may be from DMF. In the partially desolvated structure, the mask found 118 electrons per unit cell within the 732 Å^3^ of free pore volume, as expected, lower than the solvated form. However, due to the potential mixture of ethanol and water and the lower data quality of these crystals, the amount of additional solvent is indeterminate.

## 3. Characterization

**Figure 2:**
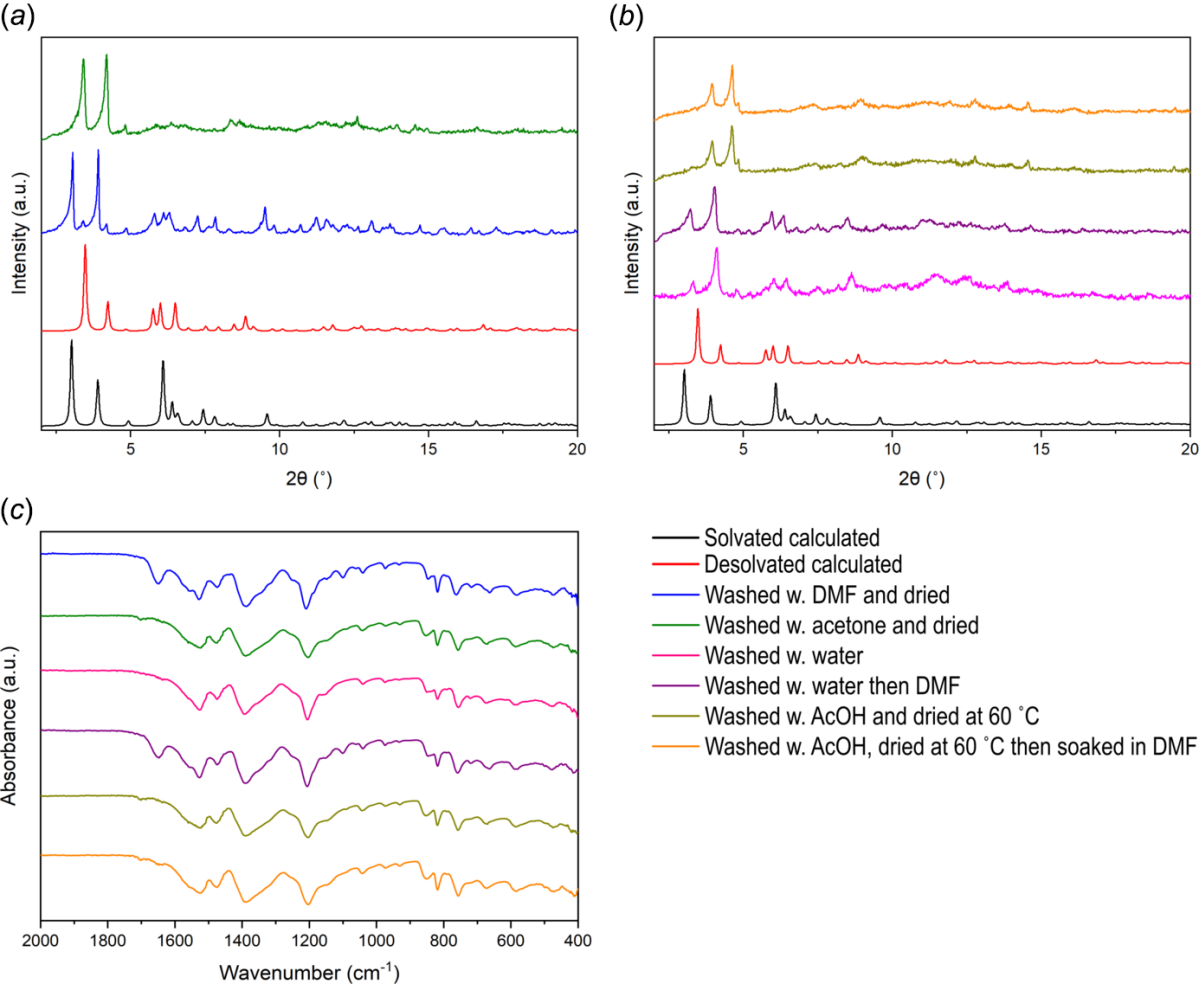
(*a*) and (*b*) PXRD patterns obtained with Mo *K*α radiation and (*c*) FTIR spectra of SIMOF-6 under different conditions: calculated solvated (black), calculated partially desolvated (red), washed with DMF (blue), washed with acetone and dried at room temperature (green), washed with water and dried (pink), then washed with DMF (purple), washed with acetone and dried at 333 K (off yellow) and then washed with DMF (orange).

The purity of SIMOF-6 synthesised in this fashion was confirmed by means of powder X-ray diffraction and FTIR spectroscopy (Fig. 2 ▸). The two phases of SIMOF-6 (solvated and partially desolvated) can both occur simultaneously. Any dry powder contains the desolvated phase, even after soaking in DMF (24 h), as can be seen in the PXRD patterns (Fig. 2 ▸*a*). The desolvated phase can be isolated by solvent exchanging with acetone (by washing on filter) and drying at room temperature and atmospheric pressure. Soaking this phase in solvent, *e.g.* DMF, can reform the solvated phase after only 5 mins of soaking. However, washing with water and then drying leads to a further phase change (Fig. 2 ▸*b*). Soaking the MOF with acetone (24 h) and drying at 333 K leads to a fourth phase with even smaller *d*-spacing (Fig. 2 ▸*b*). Neither of these phase changes can be reversed with solvent exchange and the loss in crystallinity means scXRD structure determination is not possible. FTIR shows no change in the spectra on transition between phases, with the only difference being the presence of DMF (1650 cm^−1^) in the relevant samples (Fig. 2 ▸*c*). This means that the local binding environment remains the same during phase transition with only the long-range geometry of the system changing. The high temperature drying may be removing the water mol­ecules in the ‘wall’ of the pore, thus causing a larger irreversible structural change. Removing free water may also remove these mol­ecules via capillary action causing a similar effect. Other carboxyl­ate-based Cu MOFs have shown instability to water and its removal due to the lability of the metal ion (McHugh *et al.*, 2018 ▸; Burtch *et al.*, 2014 ▸; Singh *et al.*, 2016 ▸).

Thermal gravimetric analysis of both the solvated and partially desolvated SIMOF-6 show solvent loss between 30 and 125°C, with the solvated sample showing 10 wt% more solvent loss across this temperature range (Fig  S2). Both samples start undergoing thermal decomposition at 260°C. For the solvated sample the metal linker ratio is consistent with the scXRD structure (linker/copper = 0.66). However, the desolvated sample shows a slightly reduced amount of linker (linker/copper = 0.62), perhaps suggestive of defect formation on desolvation. Furthermore, the phase change observed on complete desolvation results in low nitro­gen adsorption at 77 K (Fig.  S3) with a type II isotherm and BET surface area calculations, using the Roquerol criteria, (Rouquerol *et al.*, 2007 ▸) leading to a maximum surface area of only 6 m^2^ g^−1^, obtained after activating at 90°C, under vacuum, overnight. Even though the flexibility of the MOF means there is no measurable porosity with nitro­gen, it is clear from the crystal structure that the system is potentially porous to the right compounds.

## 4. Conclusion

We have presented here a new MOF we call SIMOF-6. It is made from five-coordinate Cu^II^ ions and 2,3-dhtp mol­ecules, which are present as both di and tetra anions. It has solvent containing 1D hexa­gonal channels and shows an inter­esting flexible behaviour in response to changes in solvation. This responsive behaviour may be useful; however, care must be taken as complete solvent removal causes an irreversible change to a dense phase.

## 5. Synthesis and crystallization

2 mmol of Cu^II^ acetate monohydrate were dissolved in 64 mL of water and 1600 µL of acetic acid. 2 mmol of 2,3-dhtp were dissolved in 64 mL of DMF. The two solutions were mixed in a glass vial and left at room temperature for 7 days. The resultant solid was separated via filtration and washed with DMF for the swollen form, or DMF, EtOH and/or acetone for the desolvated form. This produced large brown crystals of SIMOF-6.

## 6. Refinement

Crystal data, data collection and structure refinement details are summarized in Table 1 ▸. In the parent structure of SIMOF-6, non-hydrogen atoms were refined anisotropically and hydrogen atoms were refined using a riding model except the disordered aromatic hydrogens of the di-anionic 2,3-dhtp which were refined in fixed positions. To model the disordered Cu2 site, SIMU restraints of 0.02 esd were required for the metal-bound and hydrogen-bonded waters and a SIMU restraint of 0.04 esd for the metal sites. The hydrogen atoms on the disordered water were placed in calculated positions. The structure contained pores of disordered solvent (1226 Å^3^, 316 e^−^), which were treated with smtbx.mask using a 1.2 Å probe. In the structure of the partially desolvated SIMOF-6, non-hydrogen atoms were refined anisotropically, except one of the disordered phenol oxygens, which was refined isotropically due to the weaker data. Hydrogen atoms were refined using a riding model. The framework carbons were restrained with a SIMU restraint of esd 0.02 and one carbon was restrained with an ISOR restraint with esd 0.01. The metal-bound EtOH mol­ecule was subject to DFIX, DANG and SIMU restraints to maintain the expected geometry. The structure contained pores of disordered solvent (732 Å^3^, 118 e^−^), which were treated with smtbx.mask using a 1.2 Å probe.

## 7. Further characterization

Powder X-ray diffraction (PXRD) patterns were recorded on a Stoe STADI/P diffractometer using Mo *K*α_1_ radiation at room temperature in capillary Debye–Scherrer mode. FTIR spectra were obtained using a Shimadzu IRAffinity-1S spectrometer (4000-400 cm^−1^). TGA was performed using a STA780 with a crucible and a temperature ramp of 10°C min^−1^ under air flow of 30 mL min^−1^. N_2_ adsorption isotherms were recorded on a Micromeritics Tristar ii Surface Area and Porosity Instrument. Samples were added to a frit tube and activated *in vacuo* (∼3×10^−5^ mbar, 16 h) at 90°C prior to the measurement.

## Supplementary Material

Crystal structure: contains datablock(s) SIMOF6_solvated, SIMOF6_partiallydesolvated, global. DOI: 10.1107/S205698902500653X/vu2013sup1.cif

Structure factors: contains datablock(s) SIMOF6_solvated. DOI: 10.1107/S205698902500653X/vu2013SIMOF6_solvatedsup2.hkl

Structure factors: contains datablock(s) SIMOF6_partiallydesolvated. DOI: 10.1107/S205698902500653X/vu2013SIMOF6_partiallydesolvatedsup3.hkl

CCDC references: 2404309, 2404307

Additional supporting information:  crystallographic information; 3D view; checkCIF report

## Figures and Tables

**Table 1:** Experimental details

	SIMOF6 solvated	SIMOF6 partially desolvated
Crystal data		
Chemical formula	[Cu_3_(C_8_H_2_O_6_)(C_8_H_4_O_6_)(H_2_O)_3_]·H_2_O	[Cu_3_(C_8_H_2_O_6_)(C_8_H_4_O_6_)(H_2_O)_2_]·C_2_H_6_O·H_2_O
*M*_r_	217.62	680.94
Crystal system, space group	Orthorhombic, *Pnma*	Orthorhombic, *Pnma*
Temperature (K)	173	173
*a*, *b*, *c* (Å)	26.9440 (5), 16.4946 (3), 6.8910 (2)	23.4128 (16), 16.7735 (8), 7.4097 (5)
*V* (Å^3^)	3062.57 (12)	2909.9 (3)
*Z*	12	4
Radiation type	Cu *K*α	Mo *K*α
μ (mm^−1^)	2.97	2.24
Crystal size (mm)	0.03 × 0.02 × 0.02	0.06 × 0.04 × 0.02

Data collection		
Diffractometer	Rigaku XtaLAB P100K	XtaLAB AFC10 (RCD3): fixed-chi single
Absorption correction	Multi-scan (*CrysAlis PRO*; Rigaku, 2023 ▸)	Multi-scan (*CrysAlis PRO*; Rigaku, 2023 ▸)
*T*_min_, *T*_max_	0.894, 1.000	0.697, 1.000
No. of measured, independent and observed [*I* > 2σ(*I*)] reflections	26792, 2817, 2758	32110, 3725, 2739
*R*_int_	0.027	0.059
(sin θ/λ)_max_ (Å^−1^)	0.596	0.694

Refinement		
*R*[*F*^2^ > 2σ(*F*^2^)], *wR*(*F*^2^), *S*	0.047, 0.125, 1.13	0.114, 0.320, 1.08
No. of reflections	2817	3725
No. of parameters	194	198
No. of restraints	23	42
H-atom treatment	H-atom parameters constrained	H atoms treated by a mixture of independent and constrained refinement
Δρ_max_, Δρ_min_ (e Å^−3^)	1.44, −0.62	2.64, −1.16

Computer programs: *CrysAlis PRO* (Rigaku, 2023 ▸), *SHELXT2018/2* (Sheldrick, 2015*a* ▸), *SHELXL2019/3* (Sheldrick, 2015*b* ▸), *OLEX2* (Dolomanov *et al.*, 2009 ▸) and *CrystalMaker* (Palmer, 2015 ▸).

## supplementary crystallographic information

### Poly[[triaqua(µ-2,3-dihydroxyterephthalato)(µ-2,3-dioxidoterephthalato)tricopper(II)] monohydrate] (SIMOF6_solvated) Crystal data

**Table:**

[Cu_3_(C_8_H_2_O_6_)(C_8_H_4_O_6_)(H_2_O)_3_]·H_2_O	*D*_x_ = 1.416 Mg m^−^^3^
*M**_r_* = 217.62	Cu *K*α radiation, λ = 1.54184 Å
Orthorhombic, *P**n**m**a*	Cell parameters from 14159 reflections
*a* = 26.9440 (5) Å	θ = 4.2–66.3°
*b* = 16.4946 (3) Å	µ = 2.97 mm^−^^1^
*c* = 6.8910 (2) Å	*T* = 173 K
*V* = 3062.57 (12) Å^3^	Prism, orange
*Z* = 12	0.03 × 0.02 × 0.02 mm
*F*(000) = 1300	

### Poly[[triaqua(µ-2,3-dihydroxyterephthalato)(µ-2,3-dioxidoterephthalato)tricopper(II)] monohydrate] (SIMOF6_solvated) Data collection

**Table:**

Rigaku XtaLAB P100K diffractometer	2758 reflections with *I* > 2σ(*I*)
Detector resolution: 5.8140 pixels mm^-1^	*R*_int_ = 0.027
shutterless ω scans	θ_max_ = 66.8°, θ_min_ = 3.3°
Absorption correction: multi-scan (CrysAlisPro; Rigaku, 2023)	*h* = −32→31
*T*_min_ = 0.894, *T*_max_ = 1.000	*k* = −19→19
26792 measured reflections	*l* = −8→6
2817 independent reflections	

### Poly[[triaqua(µ-2,3-dihydroxyterephthalato)(µ-2,3-dioxidoterephthalato)tricopper(II)] monohydrate] (SIMOF6_solvated) Refinement

**Table:**

Refinement on *F*^2^	23 restraints
Least-squares matrix: full	Hydrogen site location: mixed
*R*[*F*^2^ > 2σ(*F*^2^)] = 0.047	H-atom parameters constrained
*wR*(*F*^2^) = 0.125	*w* = 1/[σ^2^(*F*_o_^2^) + (0.0517*P*)^2^ + 10.5953*P*] where *P* = (*F*_o_^2^ + 2*F*_c_^2^)/3
*S* = 1.13	(Δ/σ)_max_ = 0.001
2817 reflections	Δρ_max_ = 1.44 e Å^−^^3^
194 parameters	Δρ_min_ = −0.62 e Å^−^^3^

### Poly[[triaqua(µ-2,3-dihydroxyterephthalato)(µ-2,3-dioxidoterephthalato)tricopper(II)] monohydrate] (SIMOF6_solvated) Special details

**Table:**

Geometry. All esds (except the esd in the dihedral angle between two l.s. planes) are estimated using the full covariance matrix. The cell esds are taken into account individually in the estimation of esds in distances, angles and torsion angles; correlations between esds in cell parameters are only used when they are defined by crystal symmetry. An approximate (isotropic) treatment of cell esds is used for estimating esds involving l.s. planes.
Refinement. Selected crystals of as-made SIMOF-6 were analysed using a Rigaku MM-007HF High Brilliance RA generator/confocal optics with XtaLAB P100 diffractometer [Cu *K*α radiation (λ = 1.54187 Å)]. Data were collected (using a calculated strategy) and processed (including correction for Lorentz, polarization and absorption) using *CrysAlis PRO* (Rigaku Dn, 2025). Structures were solved by dual-space methods (*SHELXT2018/2*; Sheldrick, 2015*a*) and refined by full-matrix least-squares against *F*^2^ (*SHELXL2018/3*; Sheldrick, 2015*b*).

### Poly[[triaqua(µ-2,3-dihydroxyterephthalato)(µ-2,3-dioxidoterephthalato)tricopper(II)] monohydrate] (SIMOF6_solvated) Fractional atomic coordinates and isotropic or equivalent isotropic displacement parameters (Å^2^)

**Table:**

	*x*	*y*	*z*	*U*_iso_*/*U*_eq_	Occ. (<1)
Cu1	0.30580 (2)	0.56625 (3)	0.07289 (8)	0.02339 (18)	
Cu2A	0.3599 (2)	0.750000	0.0965 (14)	0.0197 (10)	0.54 (2)
O1	0.31234 (8)	0.67129 (12)	0.1972 (3)	0.0188 (5)	
O2	0.19792 (9)	0.54538 (15)	0.4762 (4)	0.0266 (6)	
O3	0.37142 (9)	0.56716 (15)	−0.0440 (4)	0.0314 (6)	
O4	0.41517 (9)	0.67057 (14)	0.0756 (4)	0.0250 (5)	
O5	0.41049 (19)	0.4260 (3)	−0.0804 (10)	0.0387 (15)	0.5
H5	0.388468	0.459996	−0.109119	0.058*	0.5
O6	0.51041 (18)	0.6622 (3)	0.0890 (10)	0.0371 (14)	0.5
H6	0.486381	0.678576	0.156045	0.056*	0.5
O7	0.25594 (9)	0.53179 (14)	0.2546 (4)	0.0316 (6)	
O8	0.26194 (12)	0.62911 (17)	−0.1621 (4)	0.0424 (7)	
H8A	0.247997	0.593000	−0.258607	0.064*	
H8B	0.281833	0.663431	−0.245261	0.064*	
O9B	0.3248 (5)	0.750000	−0.316 (3)	0.046 (3)	0.46 (2)
H9B	0.346040	0.722130	−0.374454	0.068*	0.46 (2)
C1	0.27112 (11)	0.70692 (19)	0.2709 (5)	0.0175 (6)	
C2	0.22831 (12)	0.5753 (2)	0.3604 (5)	0.0215 (7)	
C3	0.23088 (12)	0.66519 (19)	0.3492 (5)	0.0186 (7)	
C4	0.19033 (12)	0.7089 (2)	0.4263 (5)	0.0234 (7)	
H4	0.162773	0.680358	0.478614	0.028*	
C5	0.41138 (12)	0.5989 (2)	0.0134 (6)	0.0242 (7)	
C6	0.45725 (13)	0.5487 (2)	0.0037 (5)	0.0243 (7)	
C7	0.45371 (13)	0.4654 (2)	−0.0403 (6)	0.0284 (8)	
C8	0.50366 (14)	0.5821 (2)	0.0435 (6)	0.0291 (8)	
Cu2B	0.3660 (2)	0.750000	0.1545 (13)	0.0171 (10)	0.46 (2)
O9A	0.3297 (4)	0.750000	−0.218 (2)	0.030 (2)	0.54 (2)
H9A	0.312310	0.788363	−0.167610	0.044*	0.54 (2)
O10A	0.4040 (5)	0.750000	−0.435 (3)	0.149 (8)	0.54 (2)
H10A	0.386000	0.711550	−0.382607	0.223*	0.54 (2)
O10B	0.3945 (4)	0.750000	−0.5319 (17)	0.036 (3)	0.46 (2)
H10B	0.417109	0.720810	−0.474874	0.054*	0.46 (2)
H8	0.506625	0.638614	0.061442	1.000*	0.5
H7	0.421954	0.444131	−0.065336	1.000*	0.5

### Poly[[triaqua(µ-2,3-dihydroxyterephthalato)(µ-2,3-dioxidoterephthalato)tricopper(II)] monohydrate] (SIMOF6_solvated) Atomic displacement parameters (Å^2^)

**Table:**

	*U*^11^	*U*^22^	*U*^33^	*U*^12^	*U*^13^	*U*^23^
Cu1	0.0215 (3)	0.0135 (3)	0.0351 (3)	−0.00399 (18)	0.0044 (2)	−0.0071 (2)
Cu2A	0.0146 (14)	0.0086 (9)	0.036 (3)	0.000	0.0061 (15)	0.000
O1	0.0152 (11)	0.0097 (10)	0.0315 (12)	−0.0004 (8)	0.0041 (9)	0.0008 (9)
O2	0.0226 (12)	0.0170 (12)	0.0402 (14)	−0.0022 (9)	0.0058 (11)	0.0053 (11)
O3	0.0192 (12)	0.0230 (13)	0.0520 (17)	0.0023 (10)	0.0060 (11)	−0.0102 (12)
O4	0.0196 (12)	0.0136 (11)	0.0417 (15)	0.0017 (9)	0.0042 (10)	−0.0034 (10)
O5	0.017 (2)	0.020 (3)	0.078 (4)	0.009 (2)	−0.001 (3)	−0.019 (3)
O6	0.017 (2)	0.017 (2)	0.077 (4)	0.000 (2)	0.011 (3)	−0.014 (3)
O7	0.0318 (14)	0.0125 (11)	0.0505 (16)	−0.0015 (10)	0.0129 (12)	0.0004 (11)
O8	0.0565 (19)	0.0251 (14)	0.0456 (17)	−0.0024 (13)	−0.0202 (15)	0.0014 (13)
O9B	0.035 (6)	0.064 (7)	0.037 (8)	0.000	0.014 (6)	0.000
C1	0.0160 (15)	0.0139 (15)	0.0226 (16)	0.0013 (12)	−0.0018 (12)	0.0013 (13)
C2	0.0183 (16)	0.0168 (16)	0.0295 (18)	0.0000 (13)	−0.0028 (14)	0.0030 (14)
C3	0.0181 (15)	0.0151 (15)	0.0226 (16)	−0.0013 (12)	−0.0021 (13)	0.0014 (13)
C4	0.0171 (16)	0.0204 (17)	0.0327 (19)	−0.0024 (13)	0.0053 (14)	0.0031 (15)
C5	0.0206 (17)	0.0154 (16)	0.0366 (19)	−0.0007 (13)	0.0070 (15)	0.0002 (14)
C6	0.0205 (17)	0.0192 (17)	0.0334 (19)	0.0019 (14)	0.0061 (15)	−0.0042 (15)
C7	0.0243 (18)	0.0192 (17)	0.042 (2)	−0.0010 (14)	0.0050 (16)	−0.0064 (16)
C8	0.0234 (18)	0.0166 (16)	0.047 (2)	0.0000 (14)	0.0041 (16)	−0.0064 (16)
Cu2B	0.0128 (14)	0.0097 (10)	0.029 (2)	0.000	0.0026 (15)	0.000
O9A	0.042 (5)	0.026 (4)	0.021 (5)	0.000	0.009 (4)	0.000
O10A	0.037 (7)	0.37 (3)	0.040 (8)	0.000	0.017 (6)	0.000
O10B	0.027 (5)	0.057 (7)	0.025 (6)	0.000	−0.003 (4)	0.000

### Poly[[triaqua(µ-2,3-dihydroxyterephthalato)(µ-2,3-dioxidoterephthalato)tricopper(II)] monohydrate] (SIMOF6_solvated) Geometric parameters (Å, º)

**Table:**

Cu1—O1	1.941 (2)	O9B—H9B	0.838 (11)
Cu1—O2^i^	1.961 (2)	O9B—H9B^ii^	0.838 (11)
Cu1—O3	1.943 (3)	O9B—H9A^ii^	1.249 (16)
Cu1—O7	1.922 (3)	C1—C1^ii^	1.421 (6)
Cu1—O8	2.257 (3)	C1—C3	1.393 (5)
Cu2A—O1^ii^	1.952 (4)	C2—C3	1.487 (5)
Cu2A—O1	1.952 (4)	C3—C4	1.412 (5)
Cu2A—O4	1.989 (5)	C4—C4^ii^	1.357 (7)
Cu2A—O4^ii^	1.989 (5)	C4—H4	0.9500
Cu2A—O9A	2.31 (2)	C5—C6	1.489 (5)
O1—C1	1.355 (4)	C6—C7	1.410 (5)
O1—Cu2B	1.966 (5)	C6—C8	1.394 (5)
O2—C2	1.245 (4)	C7—C8^iii^	1.391 (5)
O3—C5	1.261 (4)	C7—H7	0.941 (4)
O4—C5	1.262 (4)	C8—H8	0.943 (4)
O4—Cu2B	1.940 (5)	Cu2B—O10B^iv^	2.293 (19)
O5—H5	0.8400	O9A—H9A^ii^	0.859 (7)
O5—C7	1.362 (6)	O9A—H9A	0.859 (7)
O6—H6	0.8400	O10A—H10A	0.876 (8)
O6—C8	1.370 (6)	O10A—H10A^ii^	0.876 (8)
O7—C2	1.265 (4)	O10A—H10B^ii^	0.658 (8)
O8—H8A	0.9687	O10B—H10B	0.870 (9)
O8—H8B	0.9676	O10B—H10B^ii^	0.870 (9)
			
O1—Cu1—O2^i^	173.19 (11)	O7—C2—C3	120.5 (3)
O1—Cu1—O3	95.37 (10)	C1—C3—C2	123.3 (3)
O1—Cu1—O8	87.37 (10)	C1—C3—C4	119.7 (3)
O2^i^—Cu1—O8	99.26 (11)	C4—C3—C2	117.0 (3)
O3—Cu1—O2^i^	85.01 (10)	C3—C4—H4	119.7
O3—Cu1—O8	100.11 (12)	C4^ii^—C4—C3	120.66 (19)
O7—Cu1—O1	92.29 (10)	C4^ii^—C4—H4	119.7
O7—Cu1—O2^i^	84.71 (11)	O3—C5—O4	124.4 (3)
O7—Cu1—O3	155.26 (12)	O3—C5—C6	117.6 (3)
O7—Cu1—O8	103.72 (12)	O4—C5—C6	118.0 (3)
O1^ii^—Cu2A—O1	83.4 (2)	C7—C6—C5	119.7 (3)
O1—Cu2A—O4^ii^	162.9 (6)	C8—C6—C5	121.0 (3)
O1—Cu2A—O4	94.54 (13)	C8—C6—C7	119.3 (3)
O1^ii^—Cu2A—O4	162.9 (6)	O5—C7—C6	124.5 (4)
O1^ii^—Cu2A—O4^ii^	94.54 (13)	C6—C7—H7	117.7 (4)
O1—Cu2A—O9A	95.8 (3)	C8^iii^—C7—C6	119.8 (3)
O1^ii^—Cu2A—O9A	95.9 (3)	C8^iii^—C7—H7	122.5 (4)
O4—Cu2A—O4^ii^	82.4 (3)	O6—C8—C6	123.1 (4)
O4—Cu2A—O9A	101.3 (4)	O6—C8—C7^iii^	116.0 (4)
O4^ii^—Cu2A—O9A	101.3 (4)	C6—C8—H8	119.5 (4)
Cu1—O1—Cu2A	119.8 (2)	C7^iii^—C8—C6	120.9 (3)
Cu1—O1—Cu2B	126.2 (2)	C7^iii^—C8—H8	119.3 (4)
C1—O1—Cu1	118.55 (18)	O1^ii^—Cu2B—O1	82.6 (3)
C1—O1—Cu2A	112.5 (2)	O1^ii^—Cu2B—O10B^iv^	96.0 (4)
C1—O1—Cu2B	111.9 (2)	O1—Cu2B—O10B^iv^	96.0 (4)
C2—O2—Cu1^v^	128.6 (2)	O4—Cu2B—O1^ii^	171.8 (6)
C5—O3—Cu1	130.6 (2)	O4^ii^—Cu2B—O1	171.8 (6)
C5—O4—Cu2A	125.5 (3)	O4^ii^—Cu2B—O1^ii^	95.64 (10)
C5—O4—Cu2B	132.3 (3)	O4—Cu2B—O1	95.64 (10)
C7—O5—H5	109.5	O4—Cu2B—O4^ii^	84.9 (3)
C8—O6—H6	109.5	O4^ii^—Cu2B—O10B^iv^	92.1 (3)
C2—O7—Cu1	128.2 (2)	O4—Cu2B—O10B^iv^	92.1 (3)
Cu1—O8—H8A	114.4	Cu2A—O9A—H9A	79.3 (9)
Cu1—O8—H8B	113.8	H9B^ii^—O9A—H9A^ii^	143.9 (13)
H8A—O8—H8B	99.7	H10A—O10A—H10B^ii^	179 (3)
H9B—O9B—H9B^ii^	66.6 (10)	H10A^ii^—O10A—H10B^ii^	86.5 (2)
H9B^ii^—O9B—H9A^ii^	149 (2)	Cu2B^vi^—O10B—H10A^ii^	136.5 (5)
H9B—O9B—H9A^ii^	107.4 (9)	Cu2B^vi^—O10B—H10B	131.3 (8)
O1—C1—C1^ii^	115.70 (16)	Cu2B^vi^—O10B—H10B^ii^	131.3 (8)
O1—C1—C3	124.7 (3)	H10A^ii^—O10B—H10B^ii^	57.8 (6)
C3—C1—C1^ii^	119.61 (19)	H10B—O10B—H10A^ii^	92.2 (10)
O2—C2—O7	122.1 (3)	H10B—O10B—H10B^ii^	67.2 (8)
O2—C2—C3	117.3 (3)		
			
Cu1—O1—C1—C1^ii^	−149.99 (10)	O4—C5—C6—C8	−6.2 (5)
Cu1—O1—C1—C3	32.0 (4)	O7—C2—C3—C1	−19.0 (5)
Cu1^v^—O2—C2—O7	15.5 (5)	O7—C2—C3—C4	162.2 (3)
Cu1^v^—O2—C2—C3	−164.9 (2)	C1^ii^—C1—C3—C2	−178.2 (2)
Cu1—O3—C5—O4	−47.1 (5)	C1^ii^—C1—C3—C4	0.6 (4)
Cu1—O3—C5—C6	134.1 (3)	C1—C3—C4—C4^ii^	−0.6 (4)
Cu1—O7—C2—O2	−178.4 (3)	C2—C3—C4—C4^ii^	178.3 (2)
Cu1—O7—C2—C3	2.0 (5)	C5—C6—C7—O5	1.6 (7)
Cu2A—O1—C1—C1^ii^	−3.1 (4)	C5—C6—C7—C8^iii^	−178.0 (4)
Cu2A—O1—C1—C3	178.9 (4)	C5—C6—C8—O6	−1.6 (7)
Cu2A—O4—C5—O3	−0.3 (6)	C5—C6—C8—C7^iii^	178.0 (4)
Cu2A—O4—C5—C6	178.5 (4)	C7—C6—C8—O6	−179.5 (5)
O1—C1—C3—C2	−0.3 (5)	C7—C6—C8—C7^iii^	0.0 (7)
O1—C1—C3—C4	178.4 (3)	C8—C6—C7—O5	179.6 (5)
O2—C2—C3—C1	161.4 (3)	C8—C6—C7—C8^iii^	0.0 (7)
O2—C2—C3—C4	−17.4 (5)	Cu2B—O1—C1—C1^ii^	10.5 (4)
O3—C5—C6—C7	−9.4 (5)	Cu2B—O1—C1—C3	−167.4 (4)
O3—C5—C6—C8	172.7 (4)	Cu2B—O4—C5—O3	13.3 (7)
O4—C5—C6—C7	171.7 (3)	Cu2B—O4—C5—C6	−167.8 (4)

Symmetry codes: (i) −*x*+1/2, −*y*+1, *z*−1/2; (ii) *x*, −*y*+3/2, *z*; (iii) −*x*+1, −*y*+1, −*z*; (iv) *x*, *y*, *z*+1; (v) −*x*+1/2, −*y*+1, *z*+1/2; (vi) *x*, *y*, *z*−1.

### Poly[[diaqua(µ-2,3-dihydroxyterephthalato)(µ-2,3-dioxidoterephthalato)tricopper(II)] ethanol monosolvate monohydrate] (SIMOF6_partiallydesolvated) Crystal data

**Table:**

[Cu_3_(C_8_H_2_O_6_)(C_8_H_4_O_6_)(H_2_O)_2_]·C_2_H_6_O·H_2_O	*D*_x_ = 1.554 Mg m^−^^3^
*M**_r_* = 680.94	Mo *K*α radiation, λ = 0.71073 Å
Orthorhombic, *P**n**m**a*	Cell parameters from 8615 reflections
*a* = 23.4128 (16) Å	θ = 1.8–27.8°
*b* = 16.7735 (8) Å	µ = 2.24 mm^−^^1^
*c* = 7.4097 (5) Å	*T* = 173 K
*V* = 2909.9 (3) Å^3^	Prism, orange
*Z* = 4	0.06 × 0.04 × 0.02 mm
*F*(000) = 1364	

### Poly[[diaqua(µ-2,3-dihydroxyterephthalato)(µ-2,3-dioxidoterephthalato)tricopper(II)] ethanol monosolvate monohydrate] (SIMOF6_partiallydesolvated) Data collection

**Table:**

XtaLAB AFC10 (RCD3): fixed-chi single diffractometer	2739 reflections with *I* > 2σ(*I*)
Detector resolution: 5.8140 pixels mm^-1^	*R*_int_ = 0.059
ω scans	θ_max_ = 29.6°, θ_min_ = 1.7°
Absorption correction: multi-scan (CrysAlisPro; Rigaku, 2023)	*h* = −27→30
*T*_min_ = 0.697, *T*_max_ = 1.000	*k* = −20→22
32110 measured reflections	*l* = −10→9
3725 independent reflections	

### Poly[[diaqua(µ-2,3-dihydroxyterephthalato)(µ-2,3-dioxidoterephthalato)tricopper(II)] ethanol monosolvate monohydrate] (SIMOF6_partiallydesolvated) Refinement

**Table:**

Refinement on *F*^2^	42 restraints
Least-squares matrix: full	Hydrogen site location: mixed
*R*[*F*^2^ > 2σ(*F*^2^)] = 0.114	H atoms treated by a mixture of independent and constrained refinement
*wR*(*F*^2^) = 0.320	*w* = 1/[σ^2^(*F*_o_^2^) + (0.1444*P*)^2^ + 59.2661*P*] where *P* = (*F*_o_^2^ + 2*F*_c_^2^)/3
*S* = 1.08	(Δ/σ)_max_ = 0.001
3725 reflections	Δρ_max_ = 2.64 e Å^−^^3^
198 parameters	Δρ_min_ = −1.16 e Å^−^^3^

### Poly[[diaqua(µ-2,3-dihydroxyterephthalato)(µ-2,3-dioxidoterephthalato)tricopper(II)] ethanol monosolvate monohydrate] (SIMOF6_partiallydesolvated) Special details

**Table:**

Geometry. All esds (except the esd in the dihedral angle between two l.s. planes) are estimated using the full covariance matrix. The cell esds are taken into account individually in the estimation of esds in distances, angles and torsion angles; correlations between esds in cell parameters are only used when they are defined by crystal symmetry. An approximate (isotropic) treatment of cell esds is used for estimating esds involving l.s. planes.
Refinement. *K*α radiation (λ = 1.54187 Å)]. Partially desolvated SIMOF-6 was analysed using a Rigaku FR-X Ultrahigh Brilliance Microfocus RA generator/confocal optics with XtaLAB P200 diffractometer [Mo *K*α radiation (λ = 0.71073 Å)]. Data were collected (using a calculated strategy) and processed (including correction for Lorentz, polarization and absorption) using *CrysAlis PRO* (Rigaku OD, 2025). Structures were solved by dual-space methods (*SHELXT2018/2*; Sheldrick, 2015*a*) and refined by full-matrix least-squares against *F*^2^ (*SHELXL2018/3*; Sheldrick, 2015*b*).

### Poly[[diaqua(µ-2,3-dihydroxyterephthalato)(µ-2,3-dioxidoterephthalato)tricopper(II)] ethanol monosolvate monohydrate] (SIMOF6_partiallydesolvated) Fractional atomic coordinates and isotropic or equivalent isotropic displacement parameters (Å^2^)

**Table:**

	*x*	*y*	*z*	*U*_iso_*/*U*_eq_	Occ. (<1)
Cu1	0.29605 (5)	0.56733 (6)	0.26202 (16)	0.0277 (4)	
Cu2	0.35903 (6)	0.750000	0.2711 (2)	0.0233 (4)	
O1	0.3078 (3)	0.6734 (3)	0.1571 (9)	0.0279 (14)	
O2	0.2489 (4)	0.5361 (4)	0.0619 (12)	0.048 (2)	
O3	0.2026 (3)	0.5475 (4)	−0.1936 (11)	0.0371 (17)	
O4	0.3568 (3)	0.5779 (4)	0.4383 (11)	0.0351 (16)	
O5	0.4166 (3)	0.6713 (4)	0.3352 (11)	0.0370 (17)	
O6	0.5236 (6)	0.6482 (9)	0.359 (3)	0.051 (5)	0.5
H6	0.498972	0.681979	0.388990	0.076*	0.5
O8	0.2339 (3)	0.6240 (4)	0.4564 (10)	0.0353 (15)	
H8A	0.202606	0.647098	0.397826	0.053*	
H8B	0.249220	0.668082	0.516321	0.053*	
O9	0.3092 (5)	0.750000	0.5391 (17)	0.045 (3)	
H9	0.2722 (6)	0.750000	0.551 (6)	0.068*	
C1	0.2668 (3)	0.7076 (4)	0.0569 (12)	0.0224 (16)	
C2	0.2275 (4)	0.6669 (4)	−0.0495 (12)	0.0230 (16)	
C3	0.1869 (4)	0.7092 (5)	−0.1527 (14)	0.0299 (19)	
H3	0.159445	0.680935	−0.222343	0.036*	
C4	0.2262 (4)	0.5787 (5)	−0.0615 (15)	0.032 (2)	
C5	0.4062 (4)	0.6050 (5)	0.4046 (14)	0.0302 (19)	
C6	0.4548 (4)	0.5512 (5)	0.4559 (15)	0.032 (2)	
C7	0.4437 (4)	0.4754 (6)	0.5312 (17)	0.040 (3)	
C8	0.5097 (4)	0.5760 (5)	0.4290 (16)	0.037 (2)	
C9	0.3359 (13)	0.730 (2)	0.702 (3)	0.099 (9)	0.5
H9A	0.347174	0.673833	0.687498	0.118*	0.5
H9B	0.304724	0.730425	0.792129	0.118*	0.5
C10	0.3849 (10)	0.768 (4)	0.796 (3)	0.088 (19)	0.5
H10C	0.386172	0.749199	0.921599	0.132*	0.5
H10A	0.380448	0.826005	0.794755	0.132*	0.5
H10B	0.420530	0.753350	0.735125	0.132*	0.5
O10	0.1260 (7)	0.6728 (13)	0.356 (3)	0.064 (5)	0.5
H10D	0.141075	0.719430	0.374573	0.096*	0.5
H10E	0.125126	0.650590	0.462062	0.096*	0.5
O7A	0.3929 (7)	0.4533 (13)	0.605 (4)	0.031 (6)*	0.34 (3)
O7B	0.3915 (10)	0.4416 (19)	0.525 (7)	0.015 (11)*	0.16 (3)
H7A	0.367540	0.486622	0.569610	0.023*	0.34 (3)
H7B	0.367990	0.476889	0.492511	0.023*	0.16 (3)
H8	0.520210	0.626472	0.379401	1.000*	0.5
H7	0.404706	0.460348	0.530307	1.000*	0.5

### Poly[[diaqua(µ-2,3-dihydroxyterephthalato)(µ-2,3-dioxidoterephthalato)tricopper(II)] ethanol monosolvate monohydrate] (SIMOF6_partiallydesolvated) Atomic displacement parameters (Å^2^)

**Table:**

	*U*^11^	*U*^22^	*U*^33^	*U*^12^	*U*^13^	*U*^23^
Cu1	0.0360 (7)	0.0066 (5)	0.0405 (7)	−0.0020 (4)	−0.0078 (5)	0.0031 (4)
Cu2	0.0257 (7)	0.0052 (6)	0.0391 (9)	0.000	−0.0066 (6)	0.000
O1	0.033 (3)	0.006 (2)	0.044 (4)	0.004 (2)	−0.009 (3)	0.000 (2)
O2	0.067 (5)	0.007 (3)	0.069 (5)	−0.004 (3)	−0.036 (4)	0.004 (3)
O3	0.047 (4)	0.008 (3)	0.057 (4)	0.001 (3)	0.001 (3)	−0.007 (3)
O4	0.024 (3)	0.020 (3)	0.062 (5)	−0.001 (2)	−0.011 (3)	0.013 (3)
O5	0.024 (3)	0.011 (3)	0.076 (5)	0.004 (2)	−0.012 (3)	0.007 (3)
O6	0.019 (6)	0.027 (7)	0.107 (14)	−0.013 (5)	−0.022 (8)	0.022 (9)
O8	0.031 (3)	0.020 (3)	0.054 (4)	0.002 (3)	0.004 (3)	0.001 (3)
O9	0.043 (6)	0.030 (5)	0.063 (7)	0.000	0.011 (5)	0.000
C1	0.022 (4)	0.010 (4)	0.034 (4)	0.000 (3)	−0.001 (3)	0.002 (3)
C2	0.030 (4)	0.003 (3)	0.036 (4)	0.002 (3)	0.002 (3)	0.001 (3)
C3	0.034 (4)	0.013 (4)	0.043 (5)	−0.004 (3)	−0.006 (4)	−0.002 (4)
C4	0.040 (5)	0.007 (4)	0.049 (6)	−0.001 (3)	0.005 (4)	−0.003 (4)
C5	0.026 (4)	0.013 (4)	0.051 (6)	0.000 (3)	−0.002 (4)	0.003 (4)
C6	0.023 (4)	0.012 (4)	0.062 (6)	−0.005 (3)	−0.009 (4)	0.007 (4)
C7	0.020 (4)	0.023 (5)	0.078 (8)	−0.002 (3)	−0.014 (5)	0.013 (5)
C8	0.025 (4)	0.018 (4)	0.067 (7)	−0.001 (3)	−0.007 (4)	0.007 (4)
C9	0.083 (15)	0.074 (19)	0.139 (18)	0.008 (13)	0.013 (15)	−0.010 (15)
C10	0.047 (12)	0.16 (6)	0.053 (13)	0.03 (2)	−0.002 (10)	−0.01 (2)
O10	0.030 (8)	0.088 (14)	0.075 (12)	0.016 (9)	−0.006 (8)	−0.016 (11)

### Poly[[diaqua(µ-2,3-dihydroxyterephthalato)(µ-2,3-dioxidoterephthalato)tricopper(II)] ethanol monosolvate monohydrate] (SIMOF6_partiallydesolvated) Geometric parameters (Å, º)

**Table:**

Cu1—O1	1.961 (6)	C2—C3	1.411 (12)
Cu1—O2	1.920 (7)	C2—C4	1.482 (11)
Cu1—O3^i^	1.954 (6)	C3—C3^ii^	1.369 (17)
Cu1—O4	1.939 (7)	C3—H3	0.9500
Cu1—O8	2.256 (7)	C5—C6	1.501 (12)
Cu2—O1^ii^	1.950 (6)	C6—C7	1.412 (13)
Cu2—O1	1.950 (6)	C6—C8	1.365 (13)
Cu2—O5^ii^	1.945 (6)	C7—C8^iii^	1.421 (13)
Cu2—O5	1.945 (6)	C7—O7A	1.361 (18)
Cu2—O9	2.304 (12)	C7—O7B	1.35 (2)
O1—C1	1.342 (10)	C7—H7	0.948 (9)
O2—C4	1.277 (12)	C8—H8	0.956 (9)
O3—C4	1.241 (13)	C9—H9A	0.9900
O4—C5	1.268 (11)	C9—H9B	0.9900
O5—C5	1.249 (11)	C9—C10	1.484 (19)
O6—H6	0.8400	C10—H10C	0.9800
O6—C8	1.358 (16)	C10—H10A	0.9800
O8—H8A	0.9366	C10—H10B	0.9800
O8—H8B	0.9343	O10—H10D	0.8698
O9—H9	0.870 (10)	O10—H10E	0.8700
O9—C9	1.398 (10)	O7A—H7A	0.86 (2)
C1—C1^ii^	1.422 (15)	O7B—H7B	0.84 (3)
C1—C2	1.392 (12)		
			
O1—Cu1—O8	87.8 (3)	C3—C2—C4	117.1 (8)
O2—Cu1—O1	91.3 (3)	C2—C3—H3	119.9
O2—Cu1—O3^i^	82.6 (3)	C3^ii^—C3—C2	120.2 (5)
O2—Cu1—O4	165.0 (3)	C3^ii^—C3—H3	119.9
O2—Cu1—O8	103.7 (3)	O2—C4—C2	120.5 (9)
O3^i^—Cu1—O1	163.5 (3)	O3—C4—O2	121.0 (8)
O3^i^—Cu1—O8	108.6 (3)	O3—C4—C2	118.6 (9)
O4—Cu1—O1	94.6 (3)	O4—C5—C6	115.2 (8)
O4—Cu1—O3^i^	88.0 (3)	O5—C5—O4	125.3 (8)
O4—Cu1—O8	90.2 (3)	O5—C5—C6	119.5 (8)
O1—Cu2—O1^ii^	82.4 (3)	C7—C6—C5	120.2 (8)
O1^ii^—Cu2—O9	93.5 (3)	C8—C6—C5	119.5 (8)
O1—Cu2—O9	93.5 (3)	C8—C6—C7	120.3 (8)
O5—Cu2—O1^ii^	168.2 (3)	C6—C7—C8^iii^	119.2 (8)
O5^ii^—Cu2—O1	168.2 (3)	C6—C7—H7	114.4 (9)
O5^ii^—Cu2—O1^ii^	94.9 (3)	C8^iii^—C7—H7	125.5 (9)
O5—Cu2—O1	94.9 (3)	O7A—C7—C6	124.3 (12)
O5—Cu2—O5^ii^	85.5 (4)	O7A—C7—C8^iii^	115.0 (12)
O5^ii^—Cu2—O9	98.1 (3)	O7B—C7—C6	122.1 (17)
O5—Cu2—O9	98.1 (3)	O7B—C7—C8^iii^	116.6 (16)
Cu2—O1—Cu1	120.8 (3)	O6—C8—C6	123.7 (10)
C1—O1—Cu1	120.5 (5)	C6—C8—C7^iii^	120.5 (9)
C1—O1—Cu2	113.5 (5)	C6—C8—H8	124.7 (9)
C4—O2—Cu1	129.8 (6)	C7^iii^—C8—H8	114.8 (9)
C4—O3—Cu1^iv^	122.8 (6)	O9—C9—H9A	104.6
C5—O4—Cu1	124.7 (7)	O9—C9—H9B	104.6
C5—O5—Cu2	124.7 (6)	O9—C9—C10	131 (2)
C8—O6—H6	109.5	H9A—C9—H9B	105.7
Cu1—O8—H8A	112.6	C10—C9—H9A	104.6
Cu1—O8—H8B	113.0	C10—C9—H9B	104.6
H8A—O8—H8B	101.1	C9—C10—H10C	109.5
Cu2—O9—H9	126 (3)	C9—C10—H10A	109.5
C9—O9—Cu2	121.1 (16)	C9—C10—H10B	109.5
C9—O9—H9	111 (2)	H10C—C10—H10A	109.5
O1—C1—C1^ii^	115.3 (4)	H10C—C10—H10B	109.5
O1—C1—C2	125.3 (7)	H10A—C10—H10B	109.5
C2—C1—C1^ii^	119.4 (5)	H10D—O10—H10E	104.5
C1—C2—C3	120.4 (7)	C7—O7A—H7A	107.8 (17)
C1—C2—C4	122.5 (8)	C7—O7B—H7B	108 (2)
			
Cu1—O1—C1—C1^ii^	152.4 (3)	C1^ii^—C1—C2—C3	−1.7 (10)
Cu1—O1—C1—C2	−30.5 (12)	C1^ii^—C1—C2—C4	177.8 (7)
Cu1—O2—C4—O3	171.1 (8)	C1—C2—C3—C3^ii^	1.8 (11)
Cu1—O2—C4—C2	−8.8 (15)	C1—C2—C4—O2	20.7 (15)
Cu1^iv^—O3—C4—O2	−5.8 (14)	C1—C2—C4—O3	−159.2 (9)
Cu1^iv^—O3—C4—C2	174.1 (6)	C3—C2—C4—O2	−159.7 (10)
Cu1—O4—C5—O5	55.3 (14)	C3—C2—C4—O3	20.4 (13)
Cu1—O4—C5—C6	−125.2 (8)	C4—C2—C3—C3^ii^	−177.8 (6)
Cu2—O1—C1—C1^ii^	−2.1 (6)	C5—C6—C7—C8^iii^	177.1 (10)
Cu2—O1—C1—C2	174.9 (7)	C5—C6—C7—O7A	−18 (2)
Cu2—O5—C5—O4	2.4 (15)	C5—C6—C7—O7B	14 (3)
Cu2—O5—C5—C6	−177.1 (7)	C5—C6—C8—O6	1 (2)
Cu2—O9—C9—C10	60 (4)	C5—C6—C8—C7^iii^	−177.1 (11)
O1—C1—C2—C3	−178.7 (8)	C7—C6—C8—O6	−179.5 (14)
O1—C1—C2—C4	0.9 (14)	C7—C6—C8—C7^iii^	3 (2)
O4—C5—C6—C7	1.7 (15)	C8—C6—C7—C8^iii^	−3 (2)
O4—C5—C6—C8	−178.4 (10)	C8—C6—C7—O7A	162.5 (19)
O5—C5—C6—C7	−178.8 (10)	C8—C6—C7—O7B	−166 (3)
O5—C5—C6—C8	1.2 (16)		

Symmetry codes: (i) −*x*+1/2, −*y*+1, *z*+1/2; (ii) *x*, −*y*+3/2, *z*; (iii) −*x*+1, −*y*+1, −*z*+1; (iv) −*x*+1/2, −*y*+1, *z*−1/2.
